# Estimating Cognitive Impairment in Bipolar Disorder: Should We Account for Premorbid IQ?

**DOI:** 10.1111/acps.70000

**Published:** 2025-06-18

**Authors:** Dimosthenis Tsapekos, Michail Kalfas, Rebecca Strawbridge, Samuel Swidzinski, Katherine E. Burdick, Allan H. Young

**Affiliations:** ^1^ Department of Psychological Medicine Institute of Psychiatry, Psychology and Neuroscience, King's College London London UK; ^2^ Department of Psychiatry Brigham and Women's Hospital ‐ Harvard Medical School Boston Massachusetts USA; ^3^ Division of Psychiatry, Department of Brain Sciences Imperial College London London UK

**Keywords:** bipolar, cognitive impairment, functioning, premorbid IQ

## Abstract

**Introduction:**

Cognitive impairment estimations traditionally rely on the deviation of current cognitive performance from population norms (normative approach). Since bipolar disorder (BD) has been associated with above‐average premorbid cognitive functioning in some studies, this approach might underestimate the extent of cognitive impairment in a proportion of patients. We examined whether intraindividual deviation of cognitive performance from premorbid estimates (idiographic approach) would more accurately estimate impairment in BD and assessed the functional relevance of this approach.

**Methods:**

This study pooled euthymic patients with BD (*N* = 257) taking part in two cognitive remediation trials with comparable eligibility criteria and measures. All participants underwent a baseline assessment including measures of current cognition, premorbid IQ, psychosocial functioning and mood symptom severity. We estimated and compared the prevalence of normative versus idiographic impairment and examined the association of the idiographic approach with functioning.

**Results:**

According to normative standards, 13%–31% of our euthymic sample was classified as cognitively impaired depending on the adopted cut‐off. After accounting for premorbid IQ, idiographic impairment rates increased to 37%–64%. The difference between the two approaches was significant for each cut‐off considered (0.5 and 1 SD). Idiographic cognitive impairment was significantly associated with psychosocial functioning, even after controlling for residual mood symptoms and demographic/clinical variables and functional impairment classification.

**Conclusion:**

Common practices for characterising cognitive impairment using a normative approach may underestimate the extent of impairment for a substantial proportion of euthymic patients with BD. Considering idiographic impairment provides an alternative, functionally relevant approach for cognition trials and clinical practice.

**Summary**

- Significant Outcomes
  ○ Comparisons with general population norms may underestimate the extent of cognitive difficulties experienced by a substantial proportion of euthymic people with BD.
  ○ Accounting for premorbid cognitive capacity may highlight cognitive challenges for patients who would otherwise appear cognitively intact.
  ○ This novel approach is functionally relevant, presenting a significant association with an established measure of psychosocial functioning.
- Limitations
  ○ Study participants were actively seeking treatment for cognitive impairment, which might have introduced self‐selection bias.
  ○ Premorbid cognitive ability was estimated retrospectively using a standardised measure of premorbid IQ.
  ○ Our analysis did not control for the potentially confounding effect of concomitant medications.

## 1 Introduction

Bipolar disorder (BD) is one of the most disabling psychiatric conditions, incurring a substantial burden for patients, carers, healthcare systems and society overall [1, 2]. Over the past two decades, cognitive impairment has been recognised as a prominent illness feature and a promising treatment target to improve functional capacity and reduce the burden of BD [3]. Cognitive deficits span across clinical phases and persist during euthymia [4, 5]. More importantly, these deficits are longitudinally associated with poor psychosocial functioning and quality of life [6, 7, 8].

At the population level, meta‐analytic findings suggest cognitive deficits of moderate effect size, primarily in executive functioning and verbal memory, even after controlling for potentially confounding factors, such as medication use and residual symptoms [5, 9, 10]. However, recent evidence from data‐driven classification studies has indicated a heterogeneous cognitive profile in BD, with distinct subgroups ranging from severely impaired across cognitive domains to relatively intact patients [11]. Importantly, these subgroups likely represent heterogeneous cognitive trajectories from the prodromal to the established illness phase [12].

Given that cognitive impairment is a clinically relevant component of BD and a factor hindering functional recovery, it is worth considering whether current, commonly used measurement methods accurately estimate the extent of this impairment. Evaluating cognitive impairment in people with psychiatric conditions commonly relies on performance comparisons with healthy controls or general population norms, considering the patient's age, sex and educational background; a method often referred to as the normative approach [13]. Discrepancy in either direction from the population mean is expressed in standard deviation (SD) units or percentile ranks, both enabling reliable comparisons over time and with others.

The normative approach provides a cross‐sectional estimation of the patient's impairment relative to the population's normal distribution but does not account for one's *potential*. For example, according to the normative approach, an individual with high premorbid functioning (e.g., above the 85th percentile) would be classified as ‘cognitively intact’ if their current performance is close to the 50th percentile (i.e., the mean), although this would reflect a substantial cognitive decline for that individual. Such a decline might signify cognitive challenges which would be overlooked by normative comparisons but would be captured using the idiographic approach, which provides an estimation of a patient's intraindividual changes in cognitive functioning relative to their premorbid cognitive capacity. Thus, not accounting for this capacity is likely to underestimate the extent of impairment, especially for a subgroup of BD patients with supranormal premorbid abilities [12]. This gap can be addressed by using the idiographic approach in the estimation of cognitive impairment [13].

Preliminary evidence confirms that the normative approach may underestimate the extent of cognitive impairment. Our study in euthymic BD patients (*N* = 80) showed that adjusting for premorbid IQ significantly increased rates of cognitive impairment when using cut‐offs of 0.5 and 1 SD [14]. However, no difference was found when using more conservative cut‐offs (i.e., a score of 1.5 SD or lower in the global cognitive composite), which is consistent with a study in 63 euthymic individuals [15]. At present, evidence from larger euthymic samples is lacking.

Adopting the idiographic approach may have implications for the relationship between cognitive impairment and poor psychosocial functioning. Although this association is well‐established by cross‐sectional and longitudinal research [16, 17, 18, 19, 20], there is a misalignment between the rates of cognitive and functional impairment in BD. More specifically, population‐level estimations of clinically significant cognitive impairment in euthymic patients do not exceed 40%–45% [15, 21]. In comparison, meta‐analytic estimations of functional impairment in euthymic patients reach 60% [22]. This suggests that a considerable proportion of individuals classified as ‘cognitively intact’ may still struggle functionally. Although this may be attributed to various factors (e.g., subthreshold depression, comorbidities, or sleep difficulties), it is possible that this discrepancy reflects cognitive challenges not captured by the normative approach, which contribute to functional impairment. Accurately estimating cognitive impairment in BD is crucial, as it could help us to identify those in need of cognitive interventions and to promote functional recovery in every patient with BD. As yet, the idiographic approach is not commonly applied in cognitive studies in BD, and the relationship of idiographic cognitive impairment with psychosocial functioning has not been examined in euthymic patients with BD.

### 1.1 Aims of the Study

Using a large sample of euthymic patients with BD, the aims of this study are to a) compare the rates of normative and idiographic cognitive impairment and b) examine the association of idiographic cognition with psychosocial functioning, controlling for residual mood symptoms. Based on previous findings in BD and major depression, we anticipate an increase in the prevalence of cognitive impairment after accounting for premorbid cognitive abilities (idiographic impairment), compared to the normative approach. We also hypothesise that idiographic impairment will be associated with psychosocial functioning and be predictive of functional impairment.

## 2 Materials & Methods

### 2.1 Study Design

This is a cross‐sectional secondary analysis pooling baseline data from the Cognitive Remediation in Bipolar (CRiB1 & 2) studies [23, 24], which employed comparable protocols for participant eligibility and outcome measurements. Written informed consent was obtained from all participants prior to inclusion. The CRiB1 trial was reviewed and approved by the City Road & Hampstead NHS Research Ethics Committee (15/LO/1557) and CRiB2 by the London Bromley NHS Research Ethics Committee (22/LO/0210).

### 2.2 Participants

The sample included 257 patients with a DSM‐5 diagnosis of BD (type I or type II), aged between 18 and 65 years. Participants were recruited from the community (via online advertisement and through patient organisations), as well as primary and secondary care services. The Mini International Neuropsychiatric Interview‐7 (MINI‐7) [25] was used to confirm the BD subtype. Participants had been free of acute mood symptoms for ≥ 1 month prior to inclusion, with euthymia being defined as scoring ≤ 7 on the Hamilton Depression Rating Scale 17‐item (HAMD; [26]) and the Young Mania Rating Scale (YMRS; [27]). Participants with alcohol or substance use disorder or neurological disorder were excluded.

### 2.3 Measures

Sociodemographic (e.g., age, sex, education) and clinical (e.g., BD subtype, illness duration, hospitalisations) data were collected with a structured interview. Mood symptoms were assessed using the HAMD (Hamilton, 1960) and YMRS (Young et al., 1978).

#### 2.3.1 Cognition

Cognitive assessment followed a standardised order and was conducted by a trained psychologist. For this analysis, we used tests administered in each of the two CRiB studies, assessing four cognitive domains:

1. Attention and processing speed, using the digit‐symbol coding [28];
2. Working memory, using the digit span (sum of forward, backward and sequencing conditions) [28];
3. Episodic verbal memory, using the Verbal Paired Associates II (VPA2) [29];
4. Executive functioning, using the Hotel test [30].

Raw scores from each individual test were transformed to age‐ and education‐corrected standardised scores (z scores; Mean = 0, SD = 1) according to the test manuals. Higher scores reflected better performance for all tests. A composite score for global cognition was computed by averaging individual test z scores with equal weighting. For each participant, this score represented the extent of *normative* cognitive impairment (i.e., discrepancy of current cognitive capacity from the general population average; Supplementary Table 1).

Across studies, premorbid IQ was measured using the Test of Premorbid Functioning (TOPF; [31]). The TOPF is a reliable and validated tool, with UK norms, for the estimation of intellectual capacity before illness onset. To account for this premorbid capacity, we corrected global cognition composite scores for premorbid IQ by first transforming TOPF scores to z scores and subtracting each participant's TOPF z score from their global cognition composite z score [14, 15, 32]. Estimated scores represented the extent of *idiographic* cognitive impairment for every participant (i.e., the discrepancy of current cognitive capacity from their own premorbid cognitive ability).

#### 2.3.2 Functioning

Psychosocial functioning was assessed using the Functional Assessment Short Test (FAST), a validated measure designed to evaluate functional difficulties commonly reported by people with BD [33]. FAST assessed six different domains of functioning (i.e., autonomy, occupation, cognition, financial issues, interpersonal relationships and leisure time) with higher scores representing greater levels of functional impairment. To classify functionally impaired participants, we used previously validated and clinically meaningful cut‐offs (12–20: mild impairment; 21–40: moderate impairment; > 40: severe impairment) [34].

### 2.4 Statistical Analysis

All continuous variables were checked for normality of distributions using the Shapiro–Wilk test, and log transformation was applied to conform non‐normally distributed variables. Sociodemographic and clinical characteristics were compared between the two CRiB studies using independent sample t‐tests.

To estimate the rates of normative and idiographic cognitive impairment in our sample, we compared relevant scores to pre‐defined cut‐offs classifying participants as impaired or intact. Given the lack of a consensus definition of what constitutes clinically significant cognitive impairment [35], we employed a liberal and a conservative cut‐off to define normative and idiographic impairment in line with previous research [32]: a score of −0.5 SD or lower and a score of −1 SD or lower in the two composites, respectively. For each cut‐off, we compared the rate of euthymic BD participants with normative versus idiographic impairment using paired‐samples proportion tests.

The association between the idiographic composite score and psychosocial functioning was first examined using Pearson correlation. We tested the same for the normative composite score and used Steiger's test to assess whether the strength of correlation with the FAST was significantly different between normative and idiographic impairment [36]. To further examine the relationship between idiographic cognitive impairment and functioning, we then used multiple linear regression with the FAST total score as the dependent variable and idiographic composite score as the predictor, while controlling for age, sex, education years, BD type, illness duration, previous hospitalisations and residual mood symptoms of depression (HAMD) and mania (YMRS). Pearson's Chi‐square test for categorical variables was also used to examine the relationship between the binary variables for cognitive and functional impairment. All analyses were conducted with JASP 0.19.3 [37], apart from Steiger's test, for which we used the *Coror* online calculator [38].

## 3 Results

### 3.1 Sample Characteristics

All variables met normality assumptions, and no transformation was applied. The sample had an average age of 43.5 years and primarily included female participants (70.4%). The average years of education attended was 15.9. Additional sociodemographic and clinical characteristics are presented in Table 1. The two study samples differed only in the severity of residual depressive (t = 4.265, *p* < 0.001) and manic (t = 5.866, p < 0.001) symptoms, with higher intensity for the CRiB1 study sample.

**TABLE 1:** Baseline characteristics of the sample (*N* = 257).

Variables	Mean (SD)
Age	43.5 (12.3)
Years of education	15.9 (2.4)
Illness duration (years)	12.1 (10.1)
Hospitalisations	2.2 (3.6)
HAMD	2.9 (2.3)
YMRS	1.2 (1.9)
TOPF	108.6 (8.4)
FAST	23.2 (11.1)
	*n (%)*
Sex	
Male	76 (29.6)
Female	181 (70.4)
Bipolar diagnosis	
Type I	156 (60.7)
Type II	101 (39.3)

*Note:* FAST: Functional Assessment Short Test; HAMD: Hamilton Depression Rating Scale; TOPF: Test of Premorbid Functioning; YMRS: Young Mania Rating Scale.

### 3.2 Prevalence of Normative Versus Idiographic Impairment

**FIGURE 1:**
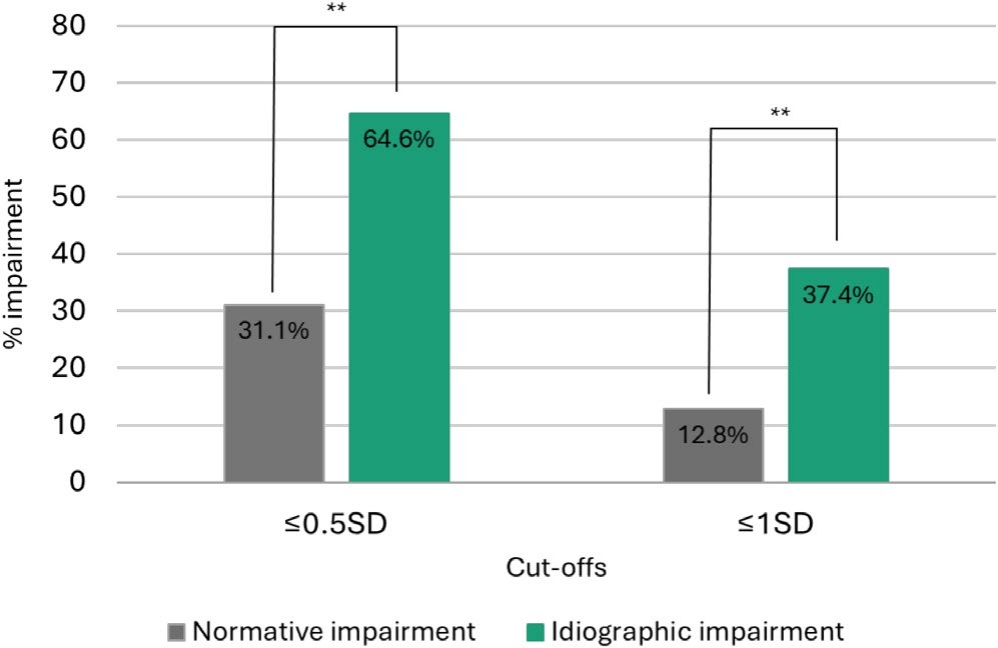
Rates and statistical comparison of normative and idiographic impairment at different cut‐offs. **p < 0.001.

Composite scores for normative and idiographic cognition were strongly correlated (*r* = 0.66, *p* < 0.001). Rates of global cognitive impairment are estimated for each approach based on these composites and comparisons between impairment rates at different cut‐offs are illustrated in Figure 1. Using normative comparisons, 31.1% (95% CI:0.25–0.37, *n* = 80) of the sample was classified as impaired according to the liberal cut‐off (≥ 0.5 SD below the normative mean), while 12.8% (95% CI:0.07–0.13, *n* = 33) presented with cognitive impairment according to the conservative cognitive impairment cut‐off (≥ 1 SD below the normative mean). When applying the idiographic impairment approach, the proportion of impaired participants increased for the 0.5 SD (64.6%, 95% CI:0.59–0.7, *n* = 166) and the 1 SD cut‐off (37.4%, 95% CI:0.31–0.43, *n* = 96).

The difference in global impairment rates after accounting for premorbid cognitive capacity was statistically significant for both cut‐offs. Specifically, we found a two‐fold increase in the rate of impairment for the 0.5 SD cut‐off (+33.4%, 95% CI: 0.27–0.39; z = 10.489, *p* < 0.001) and almost a three‐fold increase for the 1 SD cut‐off (+24.5%, 95% CI: 0.18–0.3; z = 8.163, *p* < 0.001) (Figure 1). A third of the sample (*n* = 91) appeared intact using the normative approach but was reclassified as impaired when applying the 0.5 SD idiographic impairment cut‐off. Likewise, a quarter of the normatively intact participants (*n* = 69) met the 1 SD cut‐off for idiographic impairment. The estimated premorbid IQ for the participants who were reclassified from intact to globally impaired was a full SD above the mean, independently of the cut‐off (114.3 and 115.2, respectively). In contrast, only eight participants for the 0.5 SD cut‐off and only six for the 1 SD were reclassified as intact, previously considered impaired, after considering premorbid capacity. Their premorbid IQ was estimated at half a SD below the mean for both cut‐offs (92.7 and 92.2, respectively). All impairment rates for global cognition are presented in Supplementary Table 2.

Some differences were detected when considering rates of idiographic impairment for individual domains. Taking the conservative 1 SD cut‐off, attention and processing speed showed the numerically highest impairment rate (50.2%, 95% CI:0.44–0.56, *n* = 129), followed by verbal episodic memory (47.5%, 95% CI:0.41–0.54, *n* = 122) and executive functioning (44.3%, 95% CI:0.38–0.5, *n* = 114), while working memory was idiographically impaired in one out of four participants (23.7%, 95% CI:0.18–0.24, *n* = 61).

### 3.3 Association With Psychosocial Functioning

**FIGURE 2:**
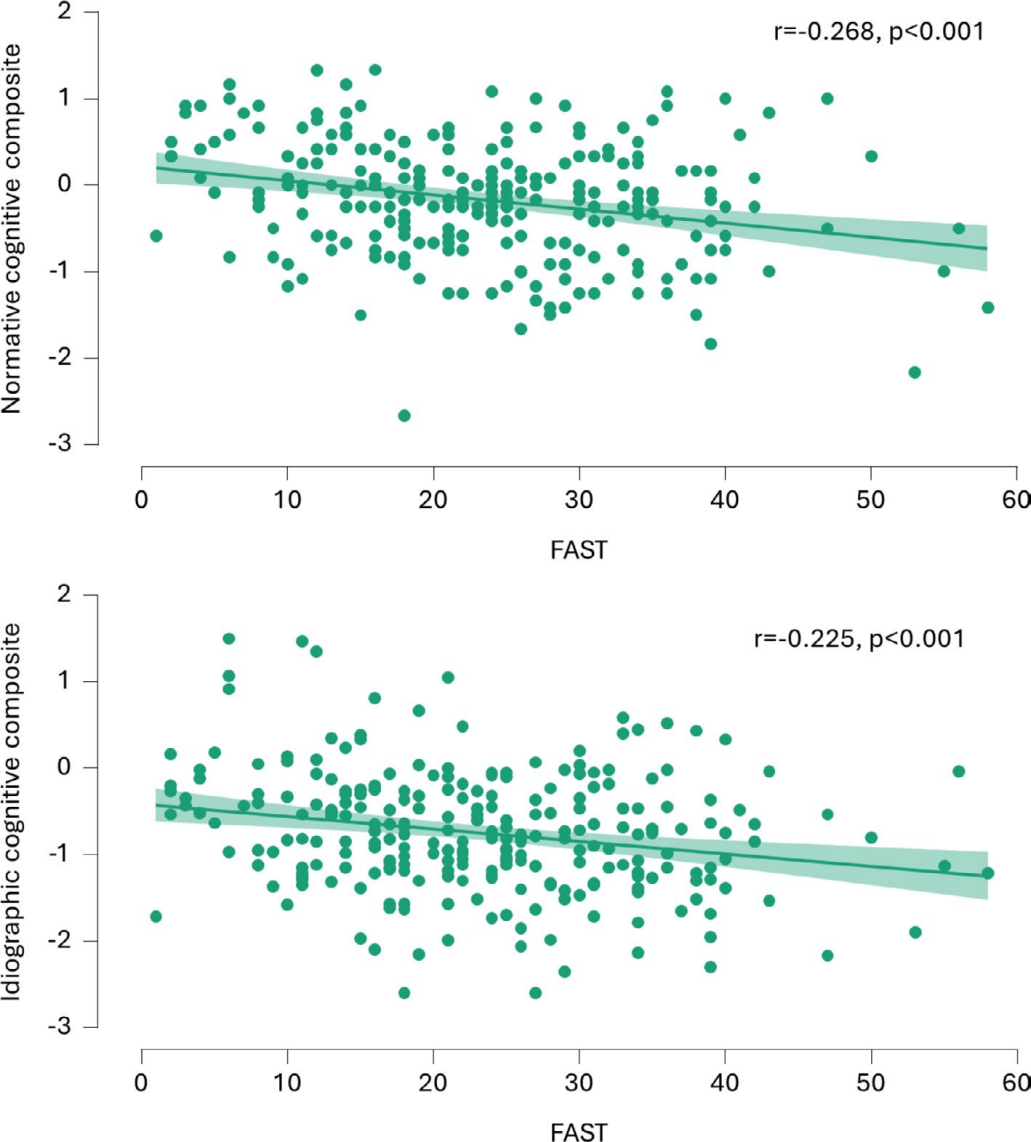
Correlation of normative and idiographic cognitive composite scores with the Functional Assessment Short Test.

Both the normative (r = −0.268, *p* < 0.001) and the idiographic (r = −0.225, *p* < 0.001) composite scores were significantly correlated with the FAST, suggesting more pronounced functional difficulties for those with lower cognitive performance and greater cognitive decline from premorbid level, respectively (Figure 2). Steiger's test indicated a relationship of equal strength with the FAST for both composite scores (z = −0.867, *p* = 0.39).

A multiple linear regression was conducted to further examine the relationship between idiographic impairment and functioning (FAST), controlling for several sociodemographic and clinical variables (Table 2). The overall model was statistically significant [F(9, 247) = 8.448, *p* < 001, adjusted R^2^ = 0.21], as was the idiographic cognitive composite predictor (beta = −0.144, *p* = 0.013). Exploratory correlations for individual cognitive domain scores showed significant correlations with functioning, with attention and processing speed (r = −0.196, *p* = 0.002) and executive functioning (r = −0.159, *p* = 0.011) showing the stronger associations.

**TABLE 2:** Multiple linear regression model for predictors of the FAST (*N* = 257).

Predictors	B^†^ (SE)	beta^‡^	p
(Intercept)	23.55 (5.02)	—	< 0.001
Age	0.20 (0.06)	0.23	**0.001**
Sex (female)	2.45 (1.37)	0.10	0.076
Years of education	−0.99 (0.26)	−0.22	**< 0.001**
HAMD	1.15 (0.28)	0.24	**< 0.001**
YMRS	0.24 (0.34)	0.04	0.480
Hospitalisations	0.21 (0.19)	0.07	0.287
Illness duration	−0.12 (0.08)	−0.11	0.138
BD type (type I)	−1.13 (1.34)	0.05	0.401
Idiographic cognitive composite	−2.25 (0.89)	−0.14	**0.013**
Model summary	*F (df)*	*R* ^*2*^ (*adjusted R* ^*2*^)	*p*
	8.448 (9,247)	0.24 (0.21)	< 0.001

*Note:* ^†^Unstandardised coefficient; ^‡^Standardised coefficient. BD: Bipolar disorder; FAST: Functional Assessment Short Test; HAMD: Hamilton Depression Rating Scale; YMRS: Young Mania Rating Scale.

Using the FAST cut‐offs, we found that 58% of the sample (95% CI:0.52–0.64, *n* = 150) were classified with moderate or severe functional impairment, while the remaining 42% experienced no or mild impairment. Cross‐tabulating the liberal 0.5 SD idiographic cut‐off with functional impairment showed a significant association between the two categorical variables (χ2 = 7.16, *p* = 0.007), indicating that being idiographically impaired predicts functional impairment classification. A comparable result was obtained when using the conservative 1 SD cut‐off (χ2 = 5.504, *p* = 0.019).

## 4 Discussion

To the best of our knowledge, this is the first study to estimate the prevalence of idiographic cognitive impairment and examine its relationship with psychosocial functioning in a large sample of euthymic patients with BD. Findings indicated that the commonly used normative approach potentially underestimates the proportion of cognitively impaired patients by around 25%–30% compared to the idiographic approach, which confirms our hypothesis. Deficits in the processing speed and verbal episodic memory domains appear to be the most frequently underestimated by the normative approach. Idiographic impairment was associated with greater functional difficulties in the presence of multiple control variables and was a significant predictor of functional impairment classification.

The rates of normative cognitive impairment (31% and 13%) identified in our pooled sample are lower compared to previous research in euthymic patients with BD [15, 21]. This is somewhat unexpected given that our sample comprised individuals actively seeking cognitive remediation therapy; however, it is possible that patients perceived changes in their cognitive abilities relative to their premorbid capacity, compelling them to seek therapy. This would be consistent with the high rates of idiographic impairment in our sample. Our analysis showed that using the normative approach, one quarter of the participants were classified as cognitively intact, despite performing at least 1 SD below what their premorbid estimates would suggest. These were all patients with high premorbid IQ (> 104), in line with previous recommendations to consider cognitive reserve to accurately estimate impairment in patients with above‐average premorbid abilities [35].

Likewise, evidence from longitudinal cohort studies suggests that increased intellectual capacity during childhood or early adulthood may represent a risk marker for developing BD in some patients [39, 40, 41]. Therefore, a significant proportion of those seeking cognitive treatment may be individuals with high premorbid functioning who have experienced a substantial decline following illness onset, despite being considered intact by normative standards. This experience of decline in cognitive skills may exacerbate a patient's subjective cognitive complaints that affect their competency in daily life activities, independently of any objective normative deficits [20, 42]. Accounting for premorbid IQ helps to capture this decline and identify those in need of cognitive interventions that would be possibly missed by normative comparisons.

The idiographic cognitive composite was significantly correlated with psychosocial functioning, with a strength of association comparable to normative cognitive impairment. This is a novel finding which provides initial validation for the functional relevance of this approach. Importantly, this association was maintained when controlled for multiple other factors potentially contributing to functioning. Although the correlation was numerically stronger for the normative composite score, this difference in strength was not significant. Considering the strong correlation between normatively and idiographically defined cognitive impairment, it is unlikely that idiographic impairment represents a distinct predictor of psychosocial functioning. These findings are in line with a similar analysis in patients with major depressive disorder, where there is evidence for an inverse relationship between functional capacity and both normative and idiographic impairment [32]. Authors also reported an association of idiographic cognition with subjectively rated functional competence, suggesting that experiencing cognitive decline may prompt a negative self‐appraisal about one's ability to perform daily life tasks. This association is yet to be tested in BD and represents an interesting question for future research.

### 4.1 Limitations

This study pooled participants from two cognitive remediation trials that shared the same eligibility criteria and administered an overlapping cognitive battery. For this study, we only considered four tests assessing core cognitive domains, and it is unclear whether a more comprehensive battery would have yielded different results. Trial participants were actively seeking treatment for cognitive impairment, which might have introduced self‐selection bias. In that sense, cognitive impairment, either normative or idiographic, could be over‐represented in our combined sample. Premorbid cognitive ability was estimated retrospectively, using a standardised measure of premorbid IQ. Other indicators of premorbid cognitive reserve were not assessed (e.g., educational attainment, occupational functioning, leisure/intellectually stimulating activities). A substantial proportion of our sample were individuals with above‐average premorbid IQ (mean = 108; 26.8% had a score > 115) which might not be representative of the overall BD population. Due to data unavailability, we did not report information on multiple clinical and illness‐related variables (e.g., age of illness onset, number of prior mood episodes, history of psychosis), which would enhance the contextualisation and generalisability of our findings. Equally, it was not possible to control for the potentially confounding effects of concomitant medications in our analysis because medication data were not accessible for participants of the ongoing CRiB2 trial. However, evidence for the impact of psychotropic medications on cognition is limited and inconsistent between studies [43].

### 4.2 Implications

In cross‐sectional examinations, we propose that the traditional normative approach to detecting cognitive impairment may underestimate the extent of cognitive challenges experienced by a substantial proportion of BD patients, who may be good candidates for cognitive interventions. Some patients seeking treatment for their cognitive difficulties may be motivated by the personal experience of progressive decline in their cognitive skills, even if not exhibiting substantial deficits according to normative standards. Clinical trials looking to enrich their sample by screening potential participants for cognitive impairment at baseline may consider incorporating a validated measure of premorbid IQ or employing another measure reflecting the participant's cognitive reserve to account for the individual variability in the progression of cognitive impairment following illness onset in BD [44]. This approach may facilitate recruitment compared to normative cognitive screening and still provide participants with ‘room‐for‐improvement’ in cognitive outcomes [14, 15]. Likewise, it is worth considering the idiographic approach in the examination of differential treatment response to cognitive treatments. Initial evidence suggests that lower baseline cognition may predict cognitive improvement following cognitive remediation therapy [45, 46]. Given that cognitive reserve may represent a marker of neuroplasticity [47, 48], future trials should examine baseline cognition as a response predictor after adjusting normative scores for premorbid IQ.

It is important to note, however, that premorbid IQ measures can only provide a proxy estimation of one's decline following illness onset. These measures are mostly reading tests that probably underestimate premorbid IQ in people with a native language other than the test itself. Hence, to increase the accuracy of these estimations and our wider understanding of the development and progression of cognitive impairment in BD, we need longitudinal studies monitoring the cognitive performance of high‐risk individuals or first‐episode patients [49]. This can also help delineate the immensely heterogeneous presentation of BD over time and characterise the factors contributing to cognitive impairment and incomplete recovery [50]. Such factors represent therapeutic targets, and their identification may enable the delivery of cognitive interventions at an earlier stage and in a targeted fashion, with the aim of preventing rather than remediating cognitive impairment.

Integrating regular assessment of cognition in clinical practice is still a work in progress [51]. Adding a measure of premorbid cognitive capacity alongside a more comprehensive cognitive battery may better inform the evaluation process and the formation of an appropriate treatment plan by setting realistic expectations, as well as identifying and prioritising those who should receive cognitive interventions.

## 5 Conclusion

Current practices of estimating cognitive impairment may underestimate the extent of cognitive difficulties experienced by a proportion of BD patients. Considering idiographic impairment with premorbid IQ measures can supplement normative comparisons and highlight challenges for patients who would otherwise appear cognitively intact. This novel approach is functionally relevant and may provide utility for cognition research and clinical practice but also requires further validation from longitudinal evidence.

## Author Contributions

D.T., R.S. and A.H.Y. conceived the study idea and planned this study. M.K. was involved in data collection. D.T. carried out the analysis and prepared the first manuscript with support from M.K. SS provided feedback from a lived experience perspective. K.E.B. and A.H.Y. supervised the project. All authors provided feedback, contributed to the final draft of the paper and approved the manuscript.

## Conflicts of Interest

M.K. declares an honorarium from Neurocentrx. R.S. declares honoraria from Lundbeck and Janssen. K.E.B. declares an honorarium from Breakthrough Discoveries for thriving with Bipolar Disorder (BD2) for her role as Chair of the Scientific Steering Committee and honoraria as a member of the scientific advisory board for Merck, Alto Neuroscience and Suven Life Sciences. A.H.Y. declares paid lectures and advisory boards for the following companies with drugs used in affective and related disorders: Flow Neuroscience, Novartis, Roche, Janssen, Takeda, Noema Pharma, Compass, AstraZenaca, Boehringer Ingelheim, Eli Lilly, LivaNova, Lundbeck, Sunovion, Servier, Allegan, Bionomics, Sumitomo Dainippon Pharma, Sage, Neurocentrx and Otsuka.

## Supporting information


**Supplementary Table 1.** Objective neuropsychological performance of the sample (*N* = 257).
**Supplementary Table 2**. Prevalence of impairment per approach and re‐classification following correction for premorbid IQ (*N* = 257).

## Data Availability

The data that support the findings of this study are available on request from the corresponding author. The data are not publicly available due to privacy or ethical restrictions.
