# Supplementary material for: Estimating Cognitive Impairment in Bipolar Disorder: Should We Account for Premorbid IQ?
Source: Acta Psychiatr Scand. 2025 Jun 18;153(5):468–76. doi: 10.1111/acps.70000 (PMC13050591; doi:10.1111/acps.70000)
Supplement: Supplementary file 1 — Supplementary Table 1. Objective neuropsychological performance of the sample (N = 257). Supplementary Table 2. Prevalence of impairment per approach and re‐classification following correction for premorbid IQ (N = 257). [file ACPS-153-468-s001.docx]

**Supplementary material**

| Supplementary Table 1. Objective neuropsychological performance of the sample (N=257). | |
| --- | --- |
| Cognitive tests | Mean (SD) |
| Digit-symbol coding | -0.36 (0.86) |
| Digit span | 0.17 (0.90) |
| Verbal paired associates II | -0.27 (1.14) |
| Hotel test | -0.22 (1.14) |
| Composite score | -0.17 (0.67) |
| *Notes:* Tests are standardized against general population norms and reported in z scores (Mean = 0, SD = 1). | |

| Supplementary Table 2. Prevalence of impairment per approach and re-classification following correction for premorbid IQ (N=257). | | |
| --- | --- | --- |
|  | **Impairment cut-offs** | |
| **Prevalence:** | **≥0.5SD** | **≥1 SD** |
| Normative impairment, n (%) | 80 (31.1) | 33 (12.8) |
| Idiographic impairment, n (%) | 166 (64.6) | 96 (37.4) |
| **Re-classification:** |  | |
| No change, n (%) | 158 (61.5) | 182 (70.8) |
| Re-classified as impaired, n (%) | 91 (35.4) | 69 (26.8) |
| Re-classified as not impaired, n (%) | 8 (3.1) | 6 (2.3) |
